# Acupuncture and weight loss in Asians: A PRISMA-compliant systematic review and meta-analysis: Erratum

**DOI:** 10.1097/MD.0000000000018400

**Published:** 2019-12-10

After a reader inquiry, there are multiple corrections being made to the Medicine article, “Acupuncture and weight loss in Asians, a PRISMA-compliant systematic review and meta-analysis,”^[1]^ published in Volume 98, Issue 33 of *Medicine*. The authors of the paper apologize for the mistakes.

1) There was an error in the Abstract Results section. The Results should read:
  Twelve RCTs involving 1151 subjects were included. Compared with the control groups, the acupuncture groups exhibited significantly greater reductions of body mass index (BMI) (WMD -1.20 kg/m^2^; 95% CI -1.91, -0.48)and waist circumference (WMD -1.85 cm; 95% CI -3.20, -0.49) In the subgroup analyses, significant differences in the reduction of BMI was observed between the acupuncture and sham acupuncture groups, the acupuncture plus diet and exercise, and the diet and exercise groups, and the acupuncture and no intervention groups, but not between the acupuncture plus exercise and exercise groups.
2) There was an error and missing reference in section 2.6 Data synthesis and analysis. The line should read “where *r* is the correlation coefficient and the value is 0.4.”[12] [43].
  References [43] Abrams KR, Gillies CL, Lambert PC. Meta-analysis of heterogeneously reported trials assessing change from baseline. Stat Med 2005; 24:3823-44.
3) There was an error in Figure 1. The “not RCT: n = 1” was missing from the figure.
4) There was an error in section 3.3 Effect of acupuncture on BMI.
  The overall efficacy of acupuncture relative to control treatment was evident from a significant difference in the reduction of BMI (WMD -1.20 kg/m^2^; 95% CI -1.91, -0.48) (Fig. 4). In the subgroup analyses, significant differences in the reduction of BMI were noted between acupuncture and sham acupuncture (WMD -0.79 kg/m^2^; 95% CI -0.99, -0.59), acupuncture plus diet and exercise and diet and exercise (WMD:-2.27 kg/m^2^; 95%CI: -4.26, -0.29) and acupuncture and no intervention(WMD:-1.70 kg/m^2^;95%CI: -2.59, -0.81). No significant differences were observed in the comparisons of acupuncture with placebo acupuncture (WMD:-0.98 kg/m^2^;95%CI: -2.26, 0.30), acupuncture plus laser acupuncture with laser acupuncture (WMD:-0.04 kg/m^2^;95%CI: -1.21, 1.13) and acupuncture plus exercise with exercise (WMD:-0.50 kg/m^2^;95%CI: -2.20, 1.20).
  Figure 4 has been updated.
5) There was an error in section 3.4 Effect of acupuncture on waist circumference.
  The overall efficacy of acupuncture relative to control treatment was evident from the significant difference in the reduction of waist circumference (WMD -1.85 cm; 95% CI -3.20, -0.49) (Fig. 5). In the subgroup analyses, there were significant differences in the reduction of waist circumference between acupuncture plus diet and exercise, and diet and exercise (WMD -4.35 cm; 95% CI -6.16, -2.54), and acupuncture and no intervention (WMD -0.29 cm; 95% CI -0.54, -0.05). There was no significant difference between acupuncture and sham acupuncture (WMD -1.28 cm; 95% CI -3.96, 1.41), acupuncture plus exercise and exercise (WMD -1.07 cm; 95% CI -4.29, 2.16).
  Figure 5 has been updated.
6) There was an error in section 4, the Discussion.
  “Our results demonstrated that relative to sham treatment, acupuncture was effective for the reduction of BMI and waist circumference.” should be revised to “Our results demonstrated that relative to sham treatment, acupuncture was effective for the reduction of BMI”

**Figure 1:**
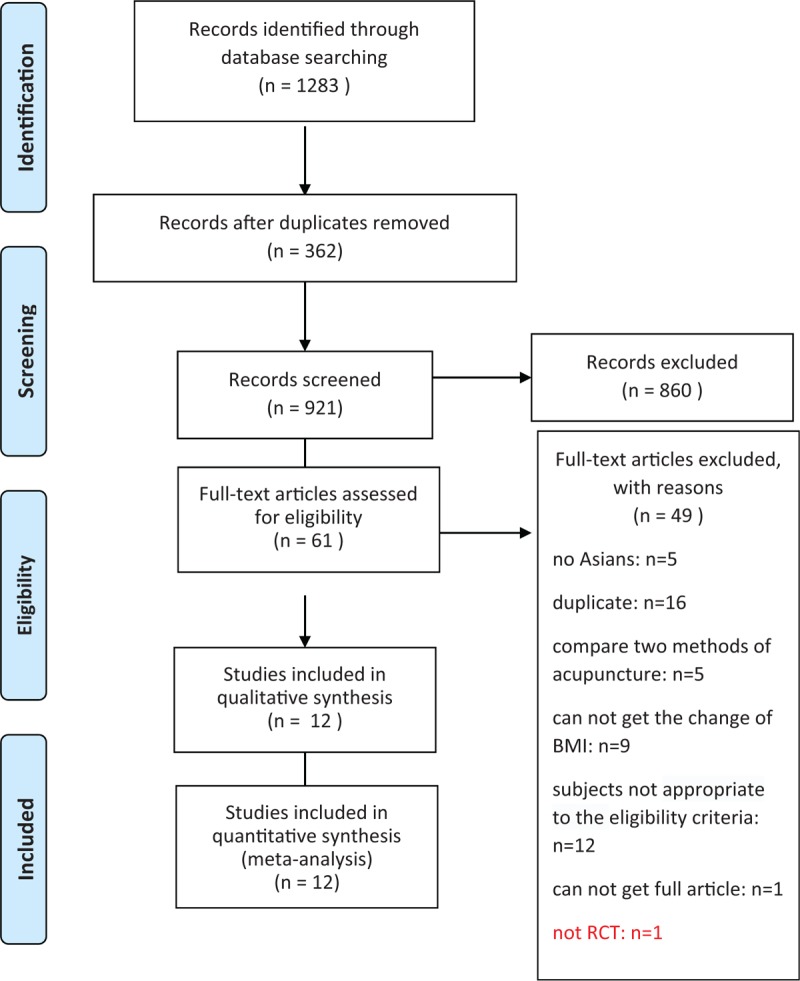
Flow diagram of study selection process.

**Figure 4:**
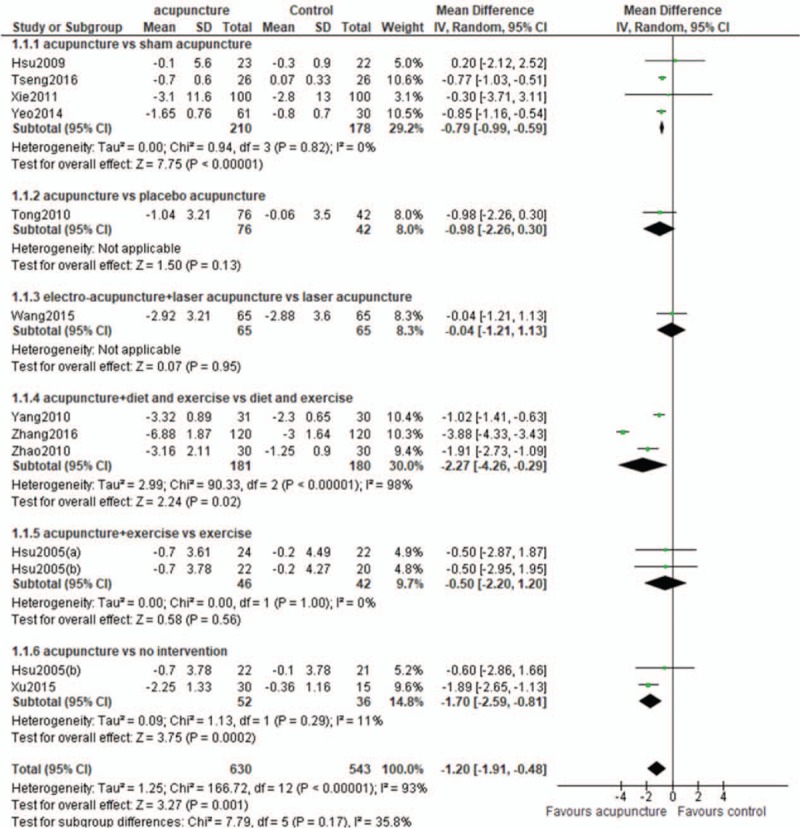
Body mass index (BMI): acupuncture vs control.

**Figure 5:**
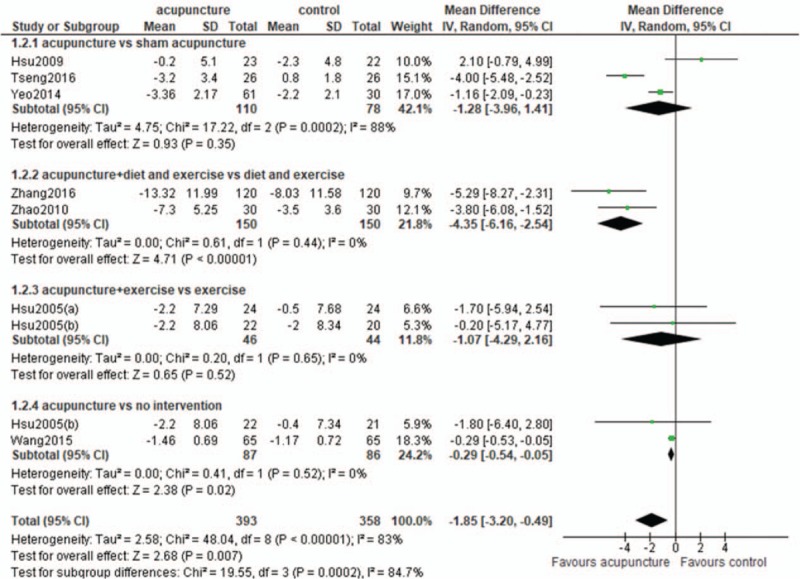
Waist circumference: acupuncture vs control.
